# Symptomatic gallstones and HIV in black South African women: Changing trends of gallstone disease?

**DOI:** 10.4102/sajhivmed.v22i1.1208

**Published:** 2021-03-25

**Authors:** Suman Mewa Kinoo, Savania Nagiah, Anil Chuturgoon, Bhugwan Singh

**Affiliations:** 1 Department of General Surgery, Faculty of Health Sciences, University of KwaZulu-Natal, Durban, South Africa; 2 Department of Human Biology, Faculty of Health Sciences, Nelson Mandela University Missionvale, Port Elizabeth, South Africa; 3 Department of Medical Biochemistry, Faculty of Health Sciences, University of KwaZulu-Natal, Durban, South Africa

**Keywords:** HIV, ART, gallstone disease, cholesterol gallstones, HIV-induced cholesterol gallstones, ARV-induced cholesterol gallstones

## Abstract

**Background:**

The incidence of metabolic disorders in human immunodeficiency virus (HIV) endemic settings is a prevailing burden in developing countries. Cholesterol homeostasis and fat metabolism are altered by HIV and antiretroviral therapy (ART), thereby possibly contributing to complications such as gallstone formation.

**Objectives:**

The aim of this study was to evaluate established risk factors for the formation of cholesterol gallstones in black South African women living with HIV (WLHIV).

**Method:**

A case series study was conducted of all black South African women undergoing cholecystectomy for gallstone disease over a 1-year period at King Edward VIII Hospital, Durban, South Africa. Age, body mass index (BMI), family history of gallstones, oestrogen exposure and lipograms were compared between WLHIV and uninfected women. Categorical variables were tested using either the Fisher’s exact test or Pearson’s chi-square test. Means were compared using independent *t*-tests. For non-normally distributed data, the Mann–Whitney U test was used. Statistical tests were two-sided, and *p*-values of less than 0.05 were considered statistically significant.

**Results:**

A total of 52 patients were assessed, 34 HIV-uninfected and 18 WLHIV. The median age of WLHIV versus the uninfected women was 35 and 50 years, respectively, (*p* = 0.015). A statistically significant number of uninfected women were in the overweight/obese category (BMI > 25 kg/m^2^) compared to the normal weight category (BMI < 25 kg/m^2^) (*p* < 0.001). The number of obese WLHIV did not reach statistical significance.

**Conclusion:**

The age of occurrence of gallstone disease amongst black South African WLHIV was significantly lower and fewer women were obese compared with the uninfected women with gallstone disease. These findings differ from known gallstone risk factors in other populations and in uninfected black South African women. This could be attributed to the metabolic alterations caused by HIV infection itself and/or to the long-term use of ART. Larger cohort studies are required to elucidate the role of HIV and ART in cholestatic disease.

## Introduction

South Africa (SA) has a population of 57.7 million. It is estimated that 7.52 million (13.1%) are people living with human immunodeficiency virus (HIV; PLWH).^1^ Close to 26% of South African PLWH reside in the province of KwaZulu-Natal. Of South African PLWH, 3.9 million (52%) are on antiretroviral therapy (ART).^1^ Most gallstone research is performed in Europe and South America. A paucity of data comes from Africa. This oversight may, in part, be because of the historically low incidence of gallstone disease (GD) amongst black South Africans,^2,3^ and/or a focus on other concurrent health crises, such as HIV, maternal death, malnutrition and other non-communicable diseases.^4^

Gallstone disease presents in a variety of ways: biliary colic, cholecystitis, gallstone pancreatitis, obstructive jaundice and as a risk for gallbladder cancer. The actual cost of GD to the already overburdened healthcare system in SA is not known. However, in developed countries, it is rated as the second highest gastrointestinal ‘cost-driver’ of the healthcare system.^5^ Gallstone disease occurs in up to 20% of the population of developed countries, but reportedly, only 5% in sub-Saharan Africa (SSA).^6^ Local reports suggest the incidence is low, but is outdated (1987).

Historically, GD was predominantly observed in South Africans of European descent. But an increase in GD in black South Africans has been noted in recent years.^3,7^ The urbanisation of black South Africans and lifestyle changes such as the consumption of high-fat, low-fibre foods, and the general rise in body mass index (BMI) have been contributory factors.^8^ This rise in GD (stones) incidence amongst black South Africans comes at a time when HIV infection has affected many and large numbers are on ART.

Human immunodeficiency virus is now a chronic, manageable disease. People living with HIV have an extended life-span. But this has come at a cost: metabolic changes in PLWH include the metabolic syndrome, type 2 diabetes mellitus, serum lipid perturbations, cardiovascular disease and changes in the distribution of body fat.^9^ Whilst there appears to be a variety of pathological processes at play, the underlying process is not identical in PLWH and the uninfected. Increased waist circumference, for instance, is less frequent in PLWH.^9^ Factors contributing to these metabolic outcomes are the host response to HIV infection, specific ART drugs, HIV-associated lipodystrophy and alcohol consumption.^10^ Genetic factors are likely to prime some in the absence of environmental factors such as smoking.^9^

As determined by cholesterol concentration, gallstones are classified into two main categories: cholesterol stones (including pure cholesterol and mixed stones) and pigment stones (black and brown stones). Cholesterol stones are the most usual and account for up to 80% of all gallstones.^11^ The pathogenesis of pigment stones is a result of infection (brown) or haemolysis (black), whereas the pathogenesis of cholesterol stones is a result of the supersaturation of cholesterol in bile.^12^

This supersaturation has been attributed to risks commonly referred to as the 5 ‘F’s (Female/oestrogen, Fat/obesity, Fertility/pregnancy, Forty/older age and Family history/genetic predisposition).^13^ People living with HIV have altered circulating lipid levels. Some changes are independent of ART and follow ongoing viral activity and the consequent inflammatory milieu within the individual, instead. In ART naïve patients with HIV-1 subtype B, very low-density lipoprotein (VLDL), cholesterol (TC) and triglycerides (TG) increase with rising HIV viral load. High-density lipoprotein (HDL) and low-density lipoprotein (LDL) decrease.^14^

African people generally display higher fasting HDL and lower TG levels than people of European descent^15^ yet HDL levels in ART-naïve PLWH in SA (HIV-1 subtype C) are low, suggesting a possible risk of CVD and possibly cholesterol-related GD.^16^

*Antiretroviral therapy* also alters lipid (cholesterol) metabolism. Protease inhibitors (PIs) generally have the greatest dyslipidaemic effect. Nucleoside reverse transcriptase inhibitors (NRTIs) and the non-nucleoside reverse transcriptase inhibitors (NNRTIs) exhibit less effect and the integrase strand transfer inhibitors (InSTIs) appear to be ‘neutral’ in this regard.^17^ In Japan, studies report an increased rate of cholelithiasis in 9.8% of PLWH possibly related to PI treatment.^18^ Lin et al. report that cumulative exposure to atazanavir/ritonavir, over a 2-year period in particular, is associated with a 6.29-fold increase in the incident-risk of cholelithiasis.^19^

*Advancing age* is a risk for gallstone formation. Aging results in greater intestinal absorption of cholesterol, greater biliary excretion of cholesterol and reduced hepatic synthesis and biliary excretion of bile salts.^20^ With ageing also comes a relative hypoperfusion of the gallbladder wall resulting in gallbladder dysfunction, together with decreased gallbladder wall contraction.^20^ All contribute to cholesterol-related GD.

*Increased weight*: The amount of cholesterol synthesised and secreted into bile is directly proportionate to weight (BMI). Overweight and the level of the BMI are directly proportional to the incidence of symptomatic gallstone per year.^21^

*Oestrogen*: Women of childbearing age, pregnant women, women on hormone replacement therapy or those with a history of high-dose oestrogen (> 50 mcg), unopposed contraception, have a 2–3 times higher risk of gallstones.^22^

*Genes:* The hypothesis of family history and GD dates back to as early as the mid-1900s.^23^ There is also growing evidence of a genetic influence.^24^ Much of the genetic studies have been undertaken in mice in which 25 lithogenic or Lith-genes have been identified.^25^

To date, there have been no studies investigating the risk factors of gallstone formation in black South African women since the early 1990s.^7^ Whether or not they conform to the same risk factors described above as in white populations is unknown. We compared well-known risk factors for cholesterol gallstone formation to validate if black South African women living with HIV (WLHIV) share the same risks as uninfected black South African women with GD. To our knowledge, this report is the first to address this issue.

## Methods

This case series study was conducted by comparing known risk factors for gallstone disease amongst black WLHIV and uninfected black South African women presenting with symptomatic gallstones requiring surgery. All patients over the age of 18 years undergoing a cholecystectomy for GD (including biliary cholic, cholecystitis, jaundice and gallstone pancreatitis) at the King Edward VIII Hospital, Durban, SA, during January to December 2017 were included in this study.

Written informed consent was received from all study participants with a standard consent form in two of the official main languages of SA (English and Zulu). The informed consent included voluntary HIV counselling and testing if this had not been assessed prior to cholecystectomy.

Clinical demographic data as per the self-identified questionnaire included sex, age, ethnicity, first degree family history of gallstones, HIV status, including the use or non-use of ART. Co-morbidity data included hypertension, diabetes mellitus and previous tuberculosis. Oestrogen exposure was defined based on pregnancies and history of contraception use and was assessed as a binary variable. Body mass index was measured by the main author using the same stadiometer for all patients in order to avoid measurement error. Patients undergoing cholecystectomy for reasons other than gallstones but where gallstones were an incidental finding were excluded from the study. Patients whose HIV status was unknown or who refused testing after voluntary counselling were also excluded. All patients had stones that macroscopically met the description of cholesterol stones. None of the patients had pigment stones. None of the patients were on any lipid-lowering medication such as fibrates which predispose to gallstones. Current viral loads (VLs) and CD4^+^ lymphocyte (CD4^+^) counts were measured in all PLWH. Lipograms (including TG, HDL, LDL and total cholesterol) were performed on all patients in a fasting state and were measured directly.

Statistical analyses were performed using R Project for Statistical Computing. Descriptive statistics such as frequencies and percentages were used to summarise categorical variables. Central tendency and dispersion of data was measured using means and standard deviations for normally distributed variables and medians and interquartile ranges (IQRs) for skewed variables. Associations between categorical variables were tested using either the Fisher’s exact test where 80% of expected cell counts < 5 or Pearson’s chi-square test where 80% of expected cell counts > 5. Similarly, with regard to the testing of associations between continuous variables: for normally distributed data, means were compared using independent *t*-tests; for non-normally distributed data, the Mann–Whitney U test was used. Statistical tests were two-sided, and *p*-values of less than 0.05 were considered as statistically significant.

### Ethical considerations

Ethics approval was granted by the University of KwaZulu-Natal’s Biomedical Research Ethics Committee (BREC), reference number BE276/16. Permission to conduct the research was granted by King Edward VIII Hospital management.

## Results

A total of 55 black South African women underwent a cholecystectomy during the study year. Three patients refused HIV testing after voluntary counselling and were excluded (*N* = 52). Median age of all women was 43 years (IQR 30–54). Patient demographic and clinical parameters are presented in Table 1. Numbers of patients on ART and ART naïve were too small to allow for any meaningful statistical comparison; therefore, only the descriptive data are presented. Clinical variables for PLWH grouped according to ART status are summarised in Table 2.

**TABLE 1:** Demographics and clinical parameters of study sample.

Baseline characteristic	HIV-negative (*N* = 34)		WLHIV (*N* = 18)		*p*
	*n*	%	*n*	%	
**Age**					
Median (Q1–Q3)	50 (31–58)	-	35 (29–42)	-	0.015
**Distribution**					
**BMI**†					
< 25	4	12	5	36	-
≥ 25	30	88	9	64	0.099
**Comorbidities**					
No	22	65	14	78	-
Yes	12	35	4	22	0.331
**Oestrogen exposure**‡					
Negative	2	6	2	14	-
Positive	29	94	12	86	0.578
**Family history**§					
None	23	74	13	93	-
Positive	8	26	1	7	0.236

Data missing for:

† , four;

‡ , seven;

§ , seven women.

BMI, body mass index; WLHIV, women living with HIV.

**TABLE 2:** Demographics and clinical parameters of women living with HIV with gallstones.

Baseline characteristic	On ART (*N* = 13)		ART naïve (*N* = 4)	
	*n*	%	*n*	%
**Age**				
Median (Q1–Q3)	38 (29–42)	-	30 (28–31)	-
**Distribution**				
**BMI**†				
< 25	4	36	1	33
≥ 25	7	64	2	67
**Comorbidities**				
No	9	69	4	100
Yes	4	31	0	0
**Oestrogen exposure**‡				
Negative	1	10	1	25
Positive	9	90	3	75
**Family history**§				
None	10	100	3	75
Positive	0	0	1	1

Data missing for:

† , three;

‡ , three;

§ , three women.

BMI, body mass index; ART, antiretroviral therapy.

**FIGURE 1:**
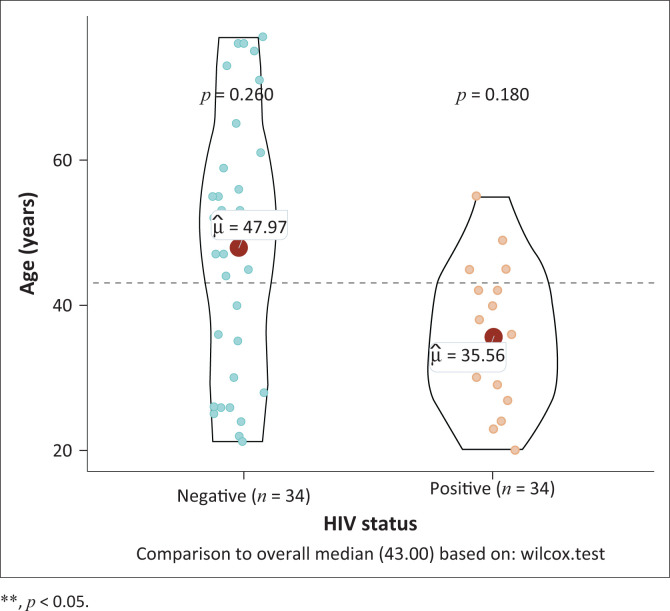
Comparative age in human immunodeficiency virus-negative individuals undergoing cholecystectomy compared to women living with human immunodeficiency virus.

Thirty-four patients were HIV-uninfected and 18 patients were PLWH. Of the 18 PLWH, 13 (72%) were on ART for an average of 4.9 years, 4 were ART naïve and 1 had missing data for ART. All patients on ART had undetectable VL, with an average CD4^+^ count of 569 cells/µL. Antiretroviral therapy naïve patients had an average VL of 89 000 copies/mL and an average CD4^+^ count of 424 cells/µL. WLHIV were younger than HIV-negative women; median ages of 35 years versus 50 years, respectively, (*p* = 0.015) (Table 1 and Figure 1).

**FIGURE 2:**
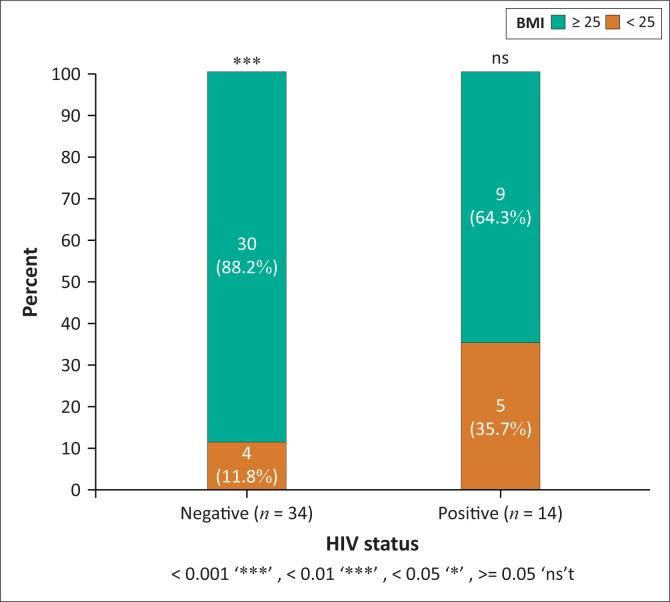
Body mass index comparison of human immunodeficiency virus-negative and women living with human immunodeficiency virus patients in the normal (< 25), overweight/obese (> 25) categories.

The median BMI of the HIV-uninfected and WLHIV was 33 kg/m^2^ (IQR 28–39), and 30 kg/m^2^ (IQR 24–35), *p* = 0.231. However, when comparing BMI within the groups stratified by HIV status, the HIV-uninfected group had a statistically significant number of patients in the overweight/obese category (BMI > 25 kg/m^2^) compared to the normal weight category, BMI > 25 kg/m^2^, *p* < 0.001. However, in the WLHIV group, differences between the weight groups were not statistically significant (Figure 2).

Seven per cent (1/14) of WLHIV compared with 26% (8/31) of HIV-uninfected women had a positive family history of gallstones (Table 1).

Oestrogen exposure was marginally higher, 94% (29/31) amongst HIV-uninfected women compared with WLHIV, 86% (12/14) (Table 1). The average use of oestrogen in the HIV-uninfected group was also greater (5 years) than the WLHIV (2 years). Hypercholesterolaemia (> 5 mmol/L) as a co-morbidity was absent in both groups of black South African women. The prevalence of overall comorbidities between the groups is shown in Table 1.

Lipogram results of patients are shown in Tables 3 and 4.

**TABLE 3:** Median lipogram values.

Parameters	Negative (*N* = 34)	WLHIV (*N* = 18)	*p*
**Total cholesterol (mmol/L)†**			
Median (Q1–Q3)	4.73 (3.74–5.51)	4.59 (4.18–5.47)	0.766
**Triglycerides (mmol/L)**‡			
Median(Q1–Q3)	1.12 (0.67–1.45)	1.04 (0.84–1.33)	0.670
**HDL (mmol/L)**§			
Median (Q1–Q3)	1.18 (0.94–1.35)	0.94 (0.91–1.32)	0.866
**LDL (mmol/L)**¶			
Median (Q1–Q3)	2.85 (2.37–3.77)	3.24 (2.93–3.74)	0.167

Data missing for:

† , four;

‡ , five;

§ , five;

¶ , six women.

LDL, low-density lipoprotein; HDL, high-density lipoprotein; WLHIV, women living with HIV.

**TABLE 4:** Percentage of patients with abnormal lipogram values.

Parameters (normal values)	Negative (*N* = 34)	Positive (*N* = 18)	*p*
**Total cholesterol group†**			
Normal (< 5 mmol/L)	19 (63%)	12 (67%)	
Abnormal	11 (37%)	6 (33%)	0.815
**Triglycerides group**‡			
Normal (< 1.7 mmol/L)	23 (79%)	15 (83%)	
Abnormal	6 (21%)	3 (17%)	1.000
**HDL group§**			
Normal (> 1.2 mmol/L)	14 (50%)	5 (29%)	
Abnormal	14 (50%)	12 (71%)	0.175
**LDL group¶**			
Normal (< 3 mmol/L)	16 (57%)	6 (35%)	
Abnormal	12 (43%)	11 (65%)	0.155

Data missing for:

† , four;

‡ , five;

§ , seven;

¶ , seven women.

LDL, low-density lipoprotein; HDL, high-density lipoprotein.

The median values of LDL were higher in the WLHIV group compared with the HIV-uninfected group; HDL levels were lower amongst WLHIV. These results did not reach statistical significance (Table 3).

The proportion of abnormal HDL and LDL levels was higher amongst WLHIV as compared with uninfected women. Differences in the groups were not statistically significant (Table 4).

## Discussion

The largest analysis of gallstone pathology in black South Africans dates to 1987, when 100 cholecystectomies were performed over a 3-year period at the Baragwanath Hospital, Johannesburg. This study demonstrated a large increase in cholecystectomies in black South Africans during the period 1967–1987. Analysis of stone and bile composition between black South Africans and people of European descent was similar.^3^ A study from the Kalafong Hospital, Pretoria, from 1988 to 2007, found the overall incidence of gallstone pancreatitis increased by 9.7% and in black South Africans by 3.4%.^26^ A further study from the King Edward VIII Hospital, Durban, SA, recorded an increase in the hospital prevalence of calculous disease in black South Africans in the 2 years after 1992 as compared with the preceding 2 years.^7^ Since 2000 however, there has been a paucity of data in the field, and perhaps, more importantly, very little that assesses the effect of the HIV epidemic and ART, on GD in PLWH.

The Swedish multigenerational registry study assessed 660 732 patients with symptomatic gallstones. The incidence per 100 000 people peaked between the ages of 30–34 years but continued to rise to ≥ 75 years confirming an increase in the incidence of gallstones with increasing age.^27^ In our study, HIV-uninfected women were more likely to be older than WLHIV. Previous reports of GD in people < 30 years attribute this age-shift to teenage pregnancy, sedentary lifestyle and a raised BMI. Of 507 patients in Rawalpindi, 48% with GD presented < 30 years. A high BMI and an elevated socioeconomic status were associated with cholelithiasis in these patients.^28^ In a large study in the United States of America, there was a comparison of patients presenting with GD in the Bronx area compared with other counties. People living with HIV and pregnant patients were excluded. Although the incidence of gallstones in patients < 20 years did not increase over the 15-year study period in counties other than the Bronx, GD in the latter increased with time. Most patients were female. The assumption at the time was that the increase was because of rising rates of teenage pregnancy and obesity in the Bronx area.^29^ The reason for young patients presenting with GD in these studies differs from our study of PLWH. The youths in these studies conformed to known risk factors. Many WLHIV in our study had normal BMIs. Oestrogen exposure from multiple pregnancies and prolonged contraception was infrequent. An alternative explanation of GB disease in our South African WLHIV is that HIV infection, its treatment or both, may be implicated.

Up to the late 1980s GD was a condition almost exclusive to people of European descent. A 1989 screening study of 100 black South African women without any gastrointestinal symptoms in Soweto, Johannesburg, nevertheless demonstrated a 10% prevalence of gallstones in the group.^2^ The BMIs of these women were significantly higher in those with gallstones. This was attributed to urbanisation and diets high in fats and low in fibre. This mirrors our findings in HIV-uninfected black South African women where a statistically significant number (88%) had a BMI > 25 kg/m^2^. There were few overweight/obese patients, BMI > 25 kg/m^2^, in the WLHIV. Rapid weight loss, as well as rapid weight gain after ART initiation in PLWH, is well documented.^30^ This is akin to weight cycling. Weight cycling is the phenomenon of intentional weight loss and regaining that weight. In patients where BMI is adjusted for, weight cycling increases the risk of gallstone formation, with larger fluctuation and more weight cycles being associated with the highest risks.^31^

Whilst normalisation of weight results in normal concentrations of cholesterol in bile, rapid weight loss, either non-operatively or operatively in the form of gastric bypass surgery, results in gallstone formation in more than 50% of patients. Mechanisms involved are increased biliary saturation secondary to increased cholesterol mobilisation, increased nucleation because of changes in bile arachidonate and glycoprotein concentrations, and elevated levels of mucin and calcium in bile.^32^ Therefore, overall BMI may not be an accurate risk stratifying tool in the development of gallstones in PLWH who may well be going through weight cycling type of phenomenon.

In a study looking at the fat distribution in PLWH at different BMIs, visceral adiposity was increased in the normal BMI group (18.5 kg/m^2^–24.9 kg/m^2^) and the overweight BMI group (25.0 kg/m^2^–29.9 kg/m^2^) relative to control subjects, but not amongst those in the obese category (≥ 30.0 kg/m^2^).^33^ There could be a link with the increase in visceral adiposity rather than to an increase in BMI to the development of gallstones in PLWH compared with uninfected patients. Furthermore, this visceral adipose tissue results in accelerated lipolysis and is what contributes to the high prevalence of non-alcoholic fatty liver disease reported in up to 30%–35% of mono-infected patients, that is, PLWH without hepatitis B or C.^34^

In the longest follow-up of body composition following ART, it was found that continued fat gain after 96 weeks of treatment was associated with the greatest risk of metabolic disorders.^35^ Southern African HIV Clinicians Society guidelines for antiretroviral therapy in adults: 2020 update,^36^ now recommend InSTI (Dolutegravir [DTG])-based therapies as the preferred first-line ART therapy. InSTIs have been touted as not affecting cholesterol metabolism which may have a big impact on preventing CVD and GD in these subsets of patients. However, there are growing reports of weight gain with the use of InSTIs,^37,38,39^ especially in black African women.^40^ One study showed a greater increase in abdominal fat compared with PIs.^41^ This is of concern considering that a large proportion of patients with CVD and GD are linked to obesity and, in particular, abdominal fat.

Familial cases account for 36% of all gallstones according to the largest family history study of GD. There is a parental family history of 50.9%, a sibling history of 35.1% and a combined parental and sibling history of 14%.^27^ The familial risk of developing gallstones is 2–4 times normal. Our HIV-uninfected group with a first degree family history of GD was 26% compared with our PLWH group which only revealed a first degree family history in 7%. This low prevalence of first degree family history of gallstones in PLWH is another risk that deviates from known risk factors of gallstone formation.

The high oestrogen levels in pregnancy are associated with increase cholesterol secretion in bile, whilst the high progesterone levels cause a decrease in bile salts and decreased contractility of the gallbladder wall resulting in precipitation of cholesterol.^22^ Males with cirrhosis of the liver have an increased incidence of gallstones because of the higher oestrogen levels.^42^ In this observational study, the overall combined oestrogen exposure rates of those patients presenting with symptomatic gallstones were 94% and 86% in the HIV-uninfected and WLHIV groups, respectively. The pregnancy rates indicated by the number of children in the WLHIV group (61%) were also lower than the HIV-uninfected group (79%). Although not statistically significant, this may point to oestrogen being less important in the WLHIV. However, the HIV-uninfected cohort would have had a longer time of oestrogen exposure and time to fall pregnant by virtue of being older compared with the WLHIV.

There is a correlation between serum total cholesterol, apolipoprotein B, C2, C3 and the amount of cholesterol saturation in bile. Also, there is a correlation with the levels of serum HDL with the amount of lecithin and bile acids in bile.^43,44^ This indicates that serum cholesterol and LDL may be a contributing factor to gallstone formation, whilst serum HDL might be protective, both of the former were markedly abnormal in our study population.

## Limitations of the study

The study is limited by its small sample size, especially with respect to WLHIV. Although we provide weight, BMI and serum lipid data, we have been unable to provide a more comprehensive evaluation of visceral adiposity. A future study will need to include abdominal fat and waist circumference measurement in the assessment. Furthermore, specific detail on antiretroviral drugs (ARVs), the regimen used and its duration, was not collected and thus linkage to an individual drug or regimen cannot be made. The case series nature of the study excludes a comparator population without GD. The lack of chemical analyses of the stones is a limitation. But pigment stones are rare, and are fairly distinctive. We believe it unlikely that these would have been missed during the pathological assessment. Nonetheless, stone analysis, particularly the exclusion of pigment stones in PLWH, may have a role in future studies.

## Conclusion

The formation of cholesterol gallstones is complex; involving genetic and environmental factors, including lifestyle. Most research into the actual cause and risk factors of gallstones has been performed in the West with a paucity of data from Africa. Whilst this has been because of the lower incidence of GD in our region, this is changing. Most black South Africans are now exposed to a Western lifestyle: urbanised diets and obesity. The patients in this study share these risks with the community. Do these risks apply equally to PLWH? From these observations, there are differences. Black South African WLHIV with GD were younger than HIV-uninfected peers. And whilst obesity and an elevated BMI were important with regard to GD in the HIV-uninfected, this was less so in WLHIV. And suggestive of a greater role of environment than genes in the development of GD in WLHIV is the relative absence of first degree relatives with the condition.
